# Evaluation of fatty acid metabolism and innate immunity interactions between commercial broiler, F1 layer × broiler cross and commercial layer strains selected for different growth potentials

**DOI:** 10.1186/s40104-017-0202-4

**Published:** 2017-09-01

**Authors:** Nicky-Lee Willson, Rebecca E. A. Forder, Rick G. Tearle, Greg S. Nattrass, Robert J. Hughes, Philip I. Hynd

**Affiliations:** 1 School of Animal and Veterinary Sciences, The University of Adelaide, Roseworthy, SA 5371 Australia; 2 Davies Research Centre, School of Animal and Veterinary Sciences, The University of Adelaide, Roseworthy, SA 5371 Australia; 3 South Australian Research and Development Institute (SARDI), Livestock and Farming Systems, Roseworthy, SA 5371 Australia; 4 South Australian Research and Development Institute (SARDI), Pig and Poultry Production Institute, Roseworthy, SA 5371 Australia; 5 The Australian Poultry and Cooperative Research Centre, University of New England, PO Box U242, Armidale, NSW 2351 Australia

**Keywords:** Broiler, Cellular stress, Fatty acid metabolism, Innate immunity, Layer, Selection

## Abstract

**Background:**

The broiler industry has undergone intense genetic selection over the past 50 yr. resulting in improvements for growth and feed efficiency, however, significant variation remains for performance and growth traits. Production improvements have been coupled with unfavourable metabolic consequences, including immunological trade-offs for growth, and excess fat deposition. To determine whether interactions between fatty acid (FA) metabolism and innate immunity may be associated with performance variations commonly seen within commercial broiler flocks, total carcass lipid %, carcass and blood FA composition, as well as genes involved with FA metabolism, immunity and cellular stress were investigated in male birds of a broiler strain, layer strain and F1 layer × broiler cross at d 14 post hatch. Heterophil: lymphocyte ratios, relative organ weights and bodyweight data were also compared.

**Results:**

Broiler bodyweight (*n* = 12) was four times that of layers (*n* = 12) by d 14 and had significantly higher carcass fat percentage compared to the cross (*n* = 6; *P* = 0.002) and layers (*P* = 0.017) which were not significantly different from each other (*P =* 0.523). The carcass and whole blood FA analysis revealed differences in the FA composition between the three groups indicating altered FA metabolism, despite all being raised on the same diet. Genes associated with FA synthesis and *β*-oxidation were upregulated in the broilers compared to the layers indicating a net overall increase in FA metabolism, which may be driven by the larger relative liver size as a percentage of bodyweight in the broilers. Genes involved in innate immunity such as *TLR2* and *TLR4*, as well as organelle stress indicators *ERN1* and *XBP1* were found to be non-significant, with the exception of high expression levels of *XBP1* in layers compared to the cross and broilers. Additionally there was no difference in heterophil: lymphocytes between any of the birds.

**Conclusions:**

The results provide evidence that genetic selection may be associated with altered metabolic processes between broilers, layers and their F1 cross. Whilst there is no evidence of interactions between FA metabolism, innate immunity or cellular stress, further investigations at later time points as growth and fat deposition increase would provide useful information as to the effects of divergent selection on key metabolic and immunological processes.

## Background

Over the past 50 yr the intensification (improved housing, husbandry and nutrition) of the broiler industry and concurrent commercial genetic selection for growth, feed efficiency and yield has resulted broiler growth increases in excess of 400% [1], with broilers having the capacity to reach 2 kg of live weight within 35 d post hatch [2, 3]. At least 85% of production improvements has been attributed to genetic selection with meat production efficiency continually increasing by 2–3% per year through selective breeding programs alone [1, 4].

Selection for feed efficiency is largely measured as feed conversion ratio (FCR), the amount of feed intake (FI) per unit bodyweight gain. In poultry systems, feed accounts for approximately 70% of total production costs [5]. Selection for efficiency has resulted in an FCR decrease of over 50% over the past 5 decades, maintaining poultry as a cost efficient source of protein [1]. Despite continued improvements, there still remains significant (>10%) variation in performance traits, including feed efficiency, bodyweight and growth rate within broiler strains [6]. This performance variation can result in an economic cost to both the producer and industry [7]. For example, variation in live weight is problematic for modern automated processing plants which reject carcasses out of a relatively narrow weight range, thus requiring further handling and sorting, and hence can incur economic loss to the processor [7].

Maintenance of innate immunity and intestinal barrier function is one parameter thought to be nutritionally costly to the host, in which exasperated or diminished immune responses could lead to increased performance variation [8]. Our previous study (Willson N-L, Nattrass GS, Hughes RJ, Forder REA, and Hynd PI, unpublished) compared high and low performing broilers to determine whether or not innate immune function could be consistently linked to the phenotypic expression of FCR. A candidate gene approach was used to determine whether functional changes in innate immune parameters could be consistently associated with high or low FCR, the results of which, there was no association. Variable expression in the pathogen recognition receptor Toll-like receptor 2 (*TLR2*) and membrane protein *CD36* also known as *FAT/CD36*, was however of interest, as both of which have been linked to each other and various roles in fatty acid metabolism. Lee and Hwang [9] have reported on links between fatty acids and TLR activation, with saturated fatty acids activating *TLR2* and *TLR4* signalling pathways and unsaturated fatty acids having an inhibitory effect on TLR-mediated signalling pathways and gene expression. *TLR2* is known to form complexes in lipid rafts with *CD36*, [10], and *CD36* has been described in facilitating *TLR2* signalling, although the mechanism remains somewhat unclear [11]. Furthermore *CD36*, is thought to promote the synthesis of triglycerides in adipocytes, the clearance of chylomicrons from plasma, as well as mediate lipid metabolism and fatty acid transport [12, 13]. Additionally, studies in broilers have found that *CD36* has a novel role in the visceral fat deposition of male broilers, and indicated that avian fat deposition has spatial and sex specific differences [14].

Fat deposition in broilers has been an unfavourable consequence of selection for growth, particularly up until the 1970s, however there has been reductions in body fat content from 26.9% in the 1970s to 15.3% in commercial breeds in the past decade (see Tallentire et al., [15] for review). Fat deposition is negatively linked to FCR, with observations that heavier chickens usually have a higher FCR and deposit a higher amount of fat [16]. The major site for fat deposition in broilers is the abdominal fat pad, which is highly correlated to total carcass fat [16, 17]. Fat has been demonstrated to account for 15–18% of the total broiler bodyweight and is considered the most variable body component, with a coefficient of variation for the total body fat content between 15 and 20%, and higher again for abdominal fat, varying between 25 and 30% [18–21]. Excess fat accumulation and the variation may be considered the net balance of dietary absorbed fat, the rate of fat synthesis (primarily hepatic lipogenesis), and fat catabolism [22]. As obesity is correlated with chronic low grade inflammation in humans [23], and that exasperated or diminished immune responses can result in inflammation potentially leading to decreased growth performance of the host, including chickens [24], it was hypothesised that interactions between fatty acid metabolism and innate immunity may be associated with performance variations commonly seen within commercial broiler flocks.

To investigate whether innate immunity and fatty acid metabolism are contributing to flock performance variation, we compared broiler and layer chicken strains that have been intensively selected for different traits; high carcass yield and growth efficiency for broilers, commercial egg production and egg efficiency for layers [25]. This selection over the years has seen the two strains diverge for these traits, with the bodyweight of broilers being five times that of layers by 6 wk of age [26]. The aim of the current experiment was to utilise broilers, layers, and a layer × broiler F1 cross to identify how genetic selection has influenced carcass lipid composition, key genes involved in fatty acid metabolism and select innate immune parameters to enable a better understanding of the biological factors underpinning feed efficiency, growth and performance variation.

## Methods

All animal procedures were approved by the University of Adelaide Animal Ethics Committee (approval #S-2015-171) and the PIRSA Animal Ethics Committee (approval #24/15).

### Birds and management

In total, 150 newly hatched male chicks (50 broiler strain, 50 F1 layer × broiler cross, 50 layer strain) were obtained from the HiChick Breeding Company Pty Ltd., Bethel, South Australia. The cross progeny were produced by HiChick utilising their commercial breeding lines. Briefly, three Isa Brown roosters and 135 Isa Brown breeder hens were used to produce layer progeny, three broiler breeder roosters and 135 broiler breeder hens used to produce the broiler progeny, and three Isa Brown roosters and 135 broiler breeder hens used to produce the F1 layer × broiler cross. All progeny were produced via natural mating. The F1 cross was utilised as an intermediate growth phenotype against broiler and layer strain progeny. Chicks were separated by breed and placed 25 chicks/rearing pen in a temperature and climate controlled room at the SARDI PPPI Poultry Research Unit, Roseworthy Campus, The University of Adelaide.

All birds were fed ad libitum (standard commercially available broiler starter diet, no in-feed antimicrobials or coccidiostats added), and had unrestricted access to water via nipple drinker lines. The three experimental groups were selected for their growth potential: Fast growing (broilers; *n* = 50) moderate growing (F1 layer × broiler; *n* = 50) and slow growing (layer strain; *n* = 50). Bodyweight was recorded weekly. On d 0, −7, −14 and −28 post hatch, 36 birds (*n* = 12 birds/breed) were randomly selected and euthanised by cervical dislocation for subsequent sampling.

### Total carcass lipid and total blood lipid composition

Eviscerated carcasses (fat pads left intact on carcass) were weighed and immediately frozen at −20 °C. Whole carcasses were submerged into liquid nitrogen for 3 min, shattered with a mallet in zip lock bags to contain all fragments, and homogenised in a 1700 W blender. Sub samples of homogenate were aliquoted (10 mL) and stored at −20 °C for analysis of total carcass lipid % and lipid composition. Total lipids were extracted at the Waite Lipid Analysis Service (WLAS), Waite Campus SA, using the methods of Folch [27]. Fatty acid composition of tissues was determined and quantified using a Hewlett-Packard 6890 GC (CA, USA) equipped with flame ionization detection and a capillary column (50 m × 0.32 mm internal diameter) coated with 70% cyanopropyl polysilphenylene-siloxane with a film thickness of 0.25 μm (BPX-70, SGE, Victoria, Australia). Fatty acid transmethylation for fatty acid methyl ester (FAME) extraction, and gas chromatography analysis of FAME were run by the methods of Folch [28]. Fatty acid peaks were identified by comparing the retention time of each peak against the retention times of a fatty acid standard of known composition. Each peak from a trace was expressed as the relative percentage of the total FAME in the sample. The detection limit of each fatty acid was 0.05% of total fatty acids.

Total blood fatty acids were measuring using the PUFAcoat dried blood spot (DBS) card, developed by the Waite Lipid Analysis Service (WLAS), Waite Campus SA. Samples were prepared by placing a drop of blood on PUFAcoat DBS card and dried at room temperature for 5 h. See Liu, Mühlhäusler [29] for full methods and validation of the PUFAcoat DBS card. In brief, lipids were extracted using a modified Folch method and FAME were extracted into heptane for gas chromatography. A Hewlett-Packard 6890 GC (CA, USA) equipped with a BPX70 capillary column 50 m × 0.32 mm, film thickness 0.25 μm (SGC Pty Ltd., Victoria, Australia), programmed temperature vaporisation injector and a flame ionisation detector (FID) was used. The identification and quantification of FAME were achieved by comparing the retention times and peak area values of unknown samples to those of commercial lipid standards (Nu-Chek Prep Inc., Elysian, MN, USA) using the Hewlett-Packard Chemstation data system.

### Heterophil: Lymphocyte ratios

Blood was collected by cardiac puncture immediately following cervical dislocation. Blood smears were made by placing 1 drop of whole blood on the end of a Starfrost frosted slide (ProSci Tech). Slides were air-dried and fixed in 100% methanol for 1 min, feather side down. Slides were stained with Geisma-Wright stain on a Hema-Tek 2000. A total of 100 cells (Cell types; lymphocytes, heterophils, eosinophils, basophils and monocytes) were counted at a 40 × magnification. Subsequent heterophil: lymphocyte ratios were determined.

### RNA extraction, library preparation and sequencing

In total, 18 liver samples from d 14 post hatch (broiler *n* = 6, cross *n* = 6, layer *n* = 6) birds were randomly selected for RNA-sequencing. Total RNA was isolated using an RNeasy Plus Mini Kit (Qiagen, Hilden, Germany). Approximately 80 mg of frozen (−80 °C) liver tissue was homogenised in 2 mL of Trizol reagent (Invitrogen, Carlsbad, CA). Aliquots of the Trizol homogenate (1 mL) were combined with chloroform (200 μL) and centrifuged for 15 mins at 4 °C. The upper aqueous phase (350 μL) was transferred to a gDNA eliminator spin column and centrifuged at >8000 × *g* (14,000 rpm) for 30 s. The flow through (300 μL) was collected and combined with 70% ethanol (300 μL) for transfer onto RNeasy columns. The remaining collection and wash steps were performed to the manufacturer’s specifications. RNA was eluted in 200 μL of RNA-free water. Purity and concentration was determined using UV spectrophotometry (Nanodrop 1000; Thermo Scienfic, Wilmington, DE).

RNA-Seq was carried out by the ACRF Cancer Genomics Facility, Adelaide, SA. The sample quality was analysed on an Agilent Bio-analyser (minimum RIN requirement of 7) and sequencing libraries were made using 2 μL of total RNA. PolyA mRNA isolation was performed using oligo dT beads. Libraries were prepared using KAPA Library Quantification Kits for Ilumina platforms (KAPABiosystems, Massachusetts, USA). 2 × 100 nt sequencing was carried out on an Illumin HiSeq 2500 Sequencing System to generate a minimum depth of 25 million reads.

### RNA sequence (RNA-seq) analysis

Reads were returned in fastq format. Low-quality base calls were trimmed from the 3′ end of reads with FastQC and adaptor sequences were trimmed from the 3′ end of reads with Cutadapt. Hisat2 [30] was used to map reads to the reference chicken genome Galgal5.0 (ftp://ftp.ncbi.nlm.nih.gov/genomes/Gallus_gallus). Duplicate reads were then removed. Stringtie [30] was used to define the transcripts from the read mappings for each sample, and to merge the transcript definitions for all samples. Transcripts were cleaned up using in-house scripts. The number of raw read counts were calculated for each transcript and sample using the function featureCounts of the R package Rsubread [31]. Another R package, edgeR [32] was used to analyse differential gene expression using normalised counts per million transcripts (CPM) to correct for varying depth of sequence among samples. Differential expression of genes was considered significant at *P* < 0.05, and a false discovery rate of <0.05, with any fold change considered. Transcript data were aggregated by gene. Genes where the maximum CPM was <1 were removed. Twenty two candidate genes primarily involved in fatty acid metabolism were selected from the RNA-seq analysis for inclusion in this study (Table 1).

**Table 1:** Candidate genes involved with fatty acid metabolism and select parameters of innate immunity

Gene name	RNA target	Accession no.^a^
*ACACA*	Acetyl-CoA Carboxylase	NM_205505.1
*ACADL*	Acyl-CoA dehydrogenase	NM_001006511.2
*ACLY*	ATP-Citrate-lyase	NM_001030540.1
*ACSL1*	Acyl-CoA synthetase	NM_001012578.1
*APOA1*	Apolipoprotein A1	NM_205525.4
*APOC3*	Apolipoprotein cIII	NM_001302127.1
*CD36*	FATCD36	NM_001030731.1
*CPT1A*	Carnitine palmitoyltransferase 1	NM_001012898.1
*CPT2*	Carnitine palmitoyltransferase 12	NM_001031287.2
*FABP1*	fatty acid binding protein 1	NM_204192.3
*FADS6*	∆6 desaturase	XM_426241.5
*FASN*	Fatty Acid Synthase	NM_205155.2
*LPL*	Lipoprotein Lipase	NM_205282.1
*MDH1*	Malate dehydrogenase	NM_001006395.2
*ME1*	Malic Enzyme 1	NM_204303.1
*PPARA*	peroxisome proliferator-activated receptor alpha	NM_001001464.1
*RXRA*	Retinoic X receptor-α	XM_003642291.3
*SCD*	Stearoyl-CoA desaturase	NM_204890.1
*TLR2A*	Toll-Like Receptor 2	NM_001161650
*TLR4*	Toll-Like Receptor-4	NM_001030693
*XBP1*	X-box binding protein	NM_001006192
*ERN1*	Inositol-requiring kinase 1	NM_001285501.1

^a^NCBI accession number

### Statistical analysis

Data were analysed by one-way ANOVA in SPSS (IBM SPSS Statistics 22). Any data not normally distributed were logged (Log_10_) to normalise and analysed by one-way ANOVA. Statistical significance was accepted at *P* < 0.05 level after which Post Hoc tests were performed using Tukeys^HSD^ to differentiate between the three groups of birds at each sampling time point.

## Results

### Bodyweight data

Bodyweights were recorded for a 28-day grow out period (Table 2). Starting bodyweights (mean ± SEM) at hatch (d 0) were significantly different between broiler *n*= 56 (44.4 ± 0.4 g); cross *n* = 57 (42.5 ± .04 g; *P* = 0.008) and layer line *n* = 54 (38.5 ± 0.4 g; *P* < 0.001) males. Bodyweights remained significantly different between all three groups of birds for the remainder of the grow-out period (*P* < 0.001).

**Table 2:** Weekly bodyweights (grams) for broiler, cross, and layer line males for d 7, −14, −21 and −28 post hatch

	d 0	d 7	d 14	d 21	d 28
Broiler	44.4 ± 0.4^a^	195 ± 2^a^	560 ± 8^a^	1153 ± 22^a^	2102 ± 35^a^
Cross	42.5 ± 0.4^b^	137 ± 3^b^	311 ± 8^b^	603 ± 12^b^	1037 ± 31^b^
Layer	38.5 ± 0.4^c^	84 ± 1^c^	159 ± 2^c^	261 ± 3.82^c^	403 ± 6^c^

^a-c^Means (± SEM) within the same column with different superscripts are significantly different (*P <* 0.05)

### Organ weights

**Fig. 1:**
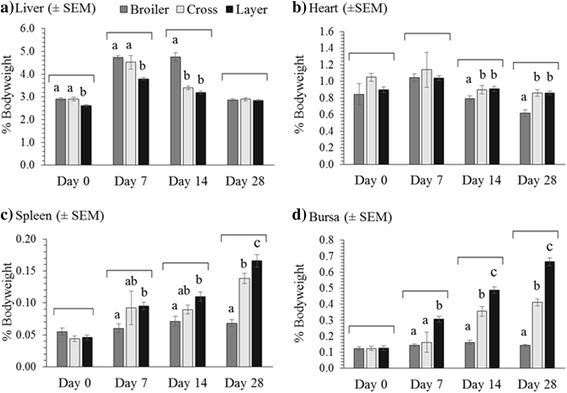
Organ weights presented as a percentage of total bodyweight (± SEM) for broiler, cross and layer line males at d 0, d 7, d 14 and d 28 post hatch for: **a**) Liver, **b**) Heart, **c**) Spleen and **d**) Bursa. *a-c* Differing scripts within each time point are significantly different (*P* < 0.05)

Organ weights were expressed as a percentage of total bodyweight to account for growth differences between broilers, layers and the F1 cross (Fig. 1). At d 0 and d 7 the layers had significantly lower relative liver weight percentages than the broiler and cross males (*P* = 0.006 and *P* < 0.001 respectively). Liver weight as a percentage of bodyweight peaked at d 14 in the broilers, which were significantly different from both the cross and layer birds (*P* < 0.001; Fig. 1a), whereas the cross and layer birds reached peak relative liver weights at d 7 post hatch. By d 28 post hatch there were no differences in relative liver weight (~2.9% of total bodyweight) between the three groups of birds (*P* = 0.852).

The heart accounted for 0.85–1.08% of total bodyweight at both d 0 and d 7 with no significant differences (*P* = 0.202 and *P =* 0.611) between broiler, cross and layers birds at each time point respectively (Fig.1b). The relative weight of the layer’s hearts remained constant for the 28 d growth period, representing ~1% of total bodyweight. The broilers had significantly lower relative heart weights than the layer and cross birds at d 14 and d 28 post hatch (*P* < 0.001).

Relative spleen weights were not different between any of the three groups at d 0 (*P* = 0.233; Fig 1c). Layers had significantly heavier relative spleen weights than broilers from d 7 post hatch onwards (*P* = 0.004). The cross and layer spleen weights continued to increase in relative weight over the 28 d period, whereas the broilers reached their maximum relative spleen weight by d 14 post hatch. By d 28 post hatch broiler spleens accounted for 0.07% of total body weight whereas layer spleens accounted for 0.17% of total bodyweight (*P* < 0.001).

No significant differences were found in relative bursa weight between broilers, layers and cross birds at d 0 (*P* = 0.997; Fig. 1d). Relative bursa weights peaked in broilers at d 14 post hatch, exhibiting a 0.04% increase from d 0-d 14 (0.12%–0.16%) then reducing slightly by d 28 to 0.14% of total bodyweight. Relative weights of the bursa increased in layer and cross birds at all sample time points. The increases were most pronounced in the layer birds with the bursa significantly different from both the crossed and layer birds at both d 14 (*P* < 0.001) and d 28 (*P* < 0.001). At d 28 post hatch the bursa weights were 0.14% and 0.67% of total bodyweight for broilers and layers respectively.

### Total carcass and total blood lipids

**Fig. 2:**
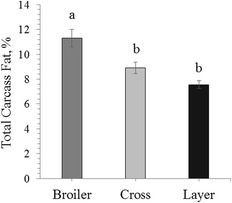
Mean ± SEM Total carcass fat % for eviscerated homogenised carcasses for broilers (*n* = 12), cross (*n* = 6) and layer line (*n* = 12) males at d 14 post hatch. *a-b* Differing scripts are statistically different (*P* < 0 .05)

Total carcass fat (%) and subsequent fatty acid composition was evaluated on eviscerated homogenised carcasses and blood samples at d 14 post hatch only. Broilers (*n* = 12) had significantly higher total carcass fat percentage (11.3%) than the cross (*n* = 6, 8.9%; *P* = 0.017) and layer line males (*n* = 12, 7.7%; *P* = 0.002; Fig. 2). The cross and layer total body fat percentages were not significantly different (*P* = 0.523).

The fatty acid composition of the carcasses varied indicating differential fatty acid metabolism (Table 3). The layers had higher levels of total saturated fatty acids (SFAs), followed by broilers, and then the cross, all significantly different (*P* = 0.001). The broilers had higher levels of palmitic acid (C16), whereas the layers had higher levels of stearic acid (C18), indicating increased elongation of SFAs in the layers. The same SFA pattern was seen in the blood (Table 4). Total carcass monounsaturated fatty acids (MUFAs) were higher in the broilers and cross relative to the layers (*P* < 0.001), indicating increased elongation of MUFAs in the broilers and cross, this pattern also reflected in the blood. The cross and layers had significantly higher carcass percentages of polyunsaturated fatty acids (PUFAs), both omega-3 and omega-6. This was reflective both the n-6: n-3 ratio as well as the PUFA: SFA ratios between the three groups of birds. The composition of the serum and the composition of the carcass was generally the same for broilers, layers and the F1 crosses.

**Table 3:** Fatty acid composition (% of total identified fatty acids) in homogenised carcass samples for broiler (*n* = 12), cross (*n* = 6) and layer line males (*n* = 12) fed the same commercial broiler diet formulation at d 14 post hatch

Fatty acid	Broiler (*n* = 12)	Cross (*n* = 6)	Layer (*n* = 12)	*P-*value
*Eviscerated carcass*				
Total Carcass Fat %	11.3^a^	8.90^b^	7.56^b^	<0.001
Total SFA	37.7 ± 0.3^b^	36.8 ± 0.2^c^	38.6 ± 0.2^a^	0.001
Palmitic acid C_16_	27.7 ± 0.24^a^	25.9 ± 0.19^b^	25.3 ± .25^b^	<0.001
Stearic acid C_18_	7.8 ± 0.12^c^	8.4 ± 0.15^b^	10.0 ± 0.18^a^	<0.001
TFA	0.8 ± 0.03^b^	0.9 ± 0.05^ab^	1.0 ± 0.06^a^	0.038
Total MUFA	49.5 ± 0.27^a^	48.7 ± 0.34^a^	44.0 ± 0.41^b^	<0.001
Palmitoleic acid (C_16_1n-7)	7.8 ± 0.17^a^	6.2 ± 0.27^b^	4.8 ± 0.19^c^	<0.001
Oleic acid (C_18_1n-9)	38.6 ± .27^a^	38.9 ± 0.27^a^	35.8 ± 0.19^b^	<0.001
Vaccenic acid (C_18_1n-7)	2.7 ± 0.07^b^	3.1 ± 0.09^a^	3.0 ± .0.06^a^	0.003
Total PUFAn-3	1.5 ± 0.01^b^	1.6 ± 0.02^b^	1.9 ± 0.05^a^	<0.001
α-Linolenic acid (C_183_n-3)	1.1 ± 0.01	1.1 ± 0.00	1.1 ± 0.01	0.684
Eicosapentanoic acid (C_22_5n-3)	0.1 ± 0.0	0.1 ± 0.0	0.1 ± 0.0	-
Docosahexanoic acid (C_22_6n-3)	0.2 ± 0.01^c^	0.3 ± 0.02^b^	0.6 ± 0.02^a^	<0.001
Total PUFAn-6	10.4 ± 0.12^c^	12.0 ± .017^b^	14.5 ± 0.32^a^	<0.001
Linoleic acid (C_18_2n-6)	9.8 ± 0.12^c^	11.0 ± 0.13^b^	12.8 ± 0.24^a^	<0.001
Arachidonic acid (C_20_4n-6)	0.3 ± 0.02^c^	0.6 ± 0.03^b^	1.1 ± 0.07^a^	<0.001
n-6: n-3 ratio	6.88^c^	7.42^b^	7.68^a^	<0.001
(MUFA + PUFA): SFA	1.61^ab^	1.68^a^	1.57^b^	0.004
PUFA: SFA	0.31^b^	0.40^a^	0.43^a^	<0.001

^1^Data are expressed as the percentage of identified fatty acids ± Standard error of means (SEM)

^a-c^Means within the same row for each parameter with different superscripts are significantly different (*P* < 0.05)

**Table 4:** Fatty acid composition (% of total identified fatty acids) in PUFAcoat DBS blood spot samples for broiler, cross and layer line males fed the same commercial broiler diet formulation at d 14 post hatch

Fatty acid	Broiler (*n* = 12)	Cross (*n* = 6)	Layer (*n* = 10)	*P*-value
Total SFA	43.7 ± 0.7	43.05 ± 0.3	46.0 ± 1.2	0.107
Palmitic acid C_16_	24. ± 0.52	22.5 ± 0.18	23.9 ± 1.78	0.424
Stearic acid C_18_	14.9 ± 0.39^b^	16.16 ± 0.21^ab^	17.07 ± 0.51^a^	0.004
TFA	0.85 ± 0.03^b^	0.93 ± 0.05^b^	1.1 ± 0.06^a^	0.004
Total MUFA	33.55 ± 0.33^a^	28.55 ± 0.65^b^	23.63 ± 0.60^c^	<0.001
Palmitoleic acid (C_16_1n-7)	4.19 ± 0.17^a^	2.55 ± 0.07^b^	1.69 ± 0.13^c^	<0.001
Oleic acid (C_18_1n-9)	26.53 ± .25^a^	23.08 ± 0.65^b^	19.36 ± 0.50^c^	<0.001
Vaccenic acid (C_18_1n-7)	1.96 ± 0.05^ab^	2.11 ± 0.06^a^	1.78 ± 0.10^b^	0.036
Total PUFAn-3	2.84 ± 0.13^b^	3.58 ± 0.18^a^	3.66 ± 0.25^a^	0.007
α-Linolenic (C_18_n-3)	0.69 ± 0.02	0.71 ± 0.03	0.63 ± 0.03	0.189
Eicosapentanoic (C_22_5n-3)	0.133 ± 0.01	0.35 ± 0.04	0.31 ± 0.03	0.628
Docosahexanoic (C_22_6n-3)	1.59 ± 0.09^b^	2.2 ± 0.14^a^	2.4 ± 0.18^a^	0.001
Total PUFAn-6	19.06 ± 0.43^v^	23.86 ± .059^b^	25.62 ± 1.1^a^	<0.001
Linoleic (C_18_2n-6)	16.38 ± 0.36^b^	19.45 ± 0.33^a^	19.72 ± 0.72^a^	<0.001
Arachidonic (C_20_4n-6)	1.26 ± 0.05^c^	2.6 ± 0.23^b^	4.06 ± 0.35^a^	<0.001
n-6: n-3 ratio	6.82	6.68	7.14	0.418
(MUFA + PUFA): SFA	1.27	1.30	1.18	0.071
PUFA: SFA	0.51^b^	0.64^a^	0.65^a^	0.002

^1^Data are expressed as the percentage of identified fatty acids ± Standard error of means (SEM)

^a-c^Means within the same row for each parameter with different superscripts are significantly different (*P* < 0.05)

### Heterophil: lymphocyte ratios

**Fig. 3:**
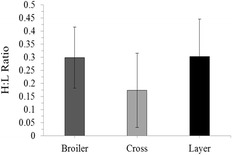
Heterophil: Lymphocyte (H:L) ratios (±SD) for broilers (*n* = 6), Cross (*n* = 6) and layer line males (*n* = 6)

The cross birds appeared to have a lower number of heterophils and a higher number of lymphocytes than the broiler and layer birds, however no statistical differences were detected in the heterophil; lymphocyte ratios between broilers, layers or the F1 cross (Fig. 3; *P* = 0.203). The differences were likely reflective of the high individual variation in cell frequencies, which is reflected by the large standard error. In addition to the heterophils and lymphocytes, basophils (*P* = 0.094), monocytes (*P* = 0.773) and eosinophils (*P* = 0.561) were also assessed however no significant differences were detected in the cell frequencies between any of the groups.

### Gene expression

**Fig. 4:**
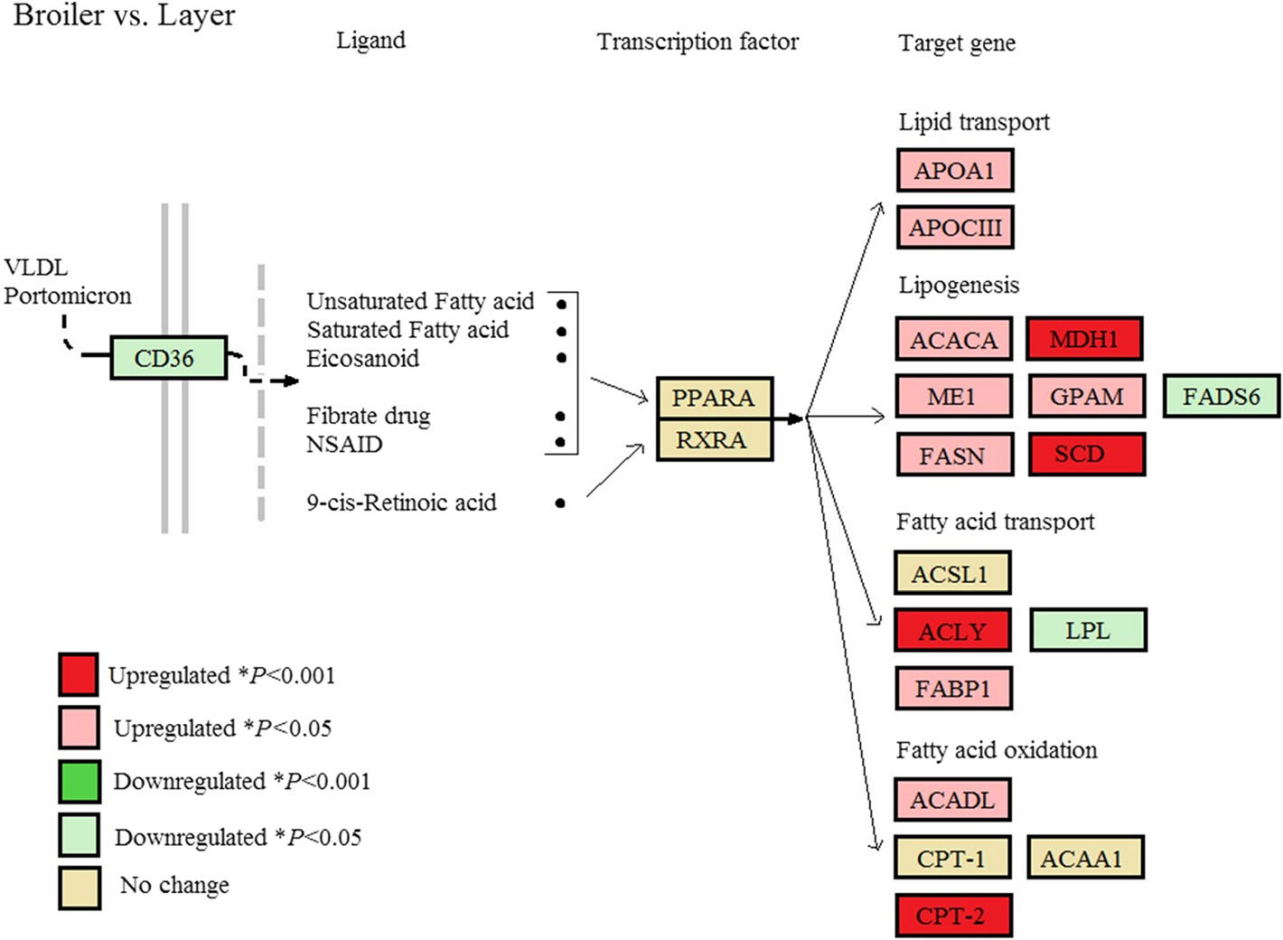
Changes in hepatic gene expression associated with the PPARA signalling pathway and fatty acid metabolism between broilers (*n* = 6) and layers (*n* = 6). *Red* boxes indicate gene upregulation in broilers, *green* boxes indicate gene downregulation in broilers in comparison to layers

The 22 candidate genes selected (Table 5) revealed that broilers (*n* = 6) in comparison to layers (*n* = 6) had significant hepatic upregulation of genes involved in lipid transport; *APOA1* (*P* = 0.019)*, APOC3* (*P* = 0.003), lipogenesis; *ACACA* (*P* = 0.001)*, ME1* (*P* = 0.022)*, FASN* (*P* < 0.001)*, GPAM* (*P* = 0.001), *MDH1* (*P* < 0.001)*, SCD* (*P* < 0.001), fatty acid transport; *FABP1* (*P* = 0.001), *ACLY* (*P* < 0.001), and fatty acid oxidation; *ACADL* (*P* = 0.003), *CPT-2* (*P* < 0.001), (Fig. 4). An exception was the down-regulation of *FADS6* (*P* = 0.054) in broilers, a rate-limiting enzyme involved in the elongation of PUFAs. Broilers when compared to the cross (*n* = 6) birds exhibited generalised upregulation of fatty acid metabolism, although not as pronounced as seen between broilers and layers. Significant hepatic upregulation for lipid transport; *APOC3* (*P* = 0.029), lipogenesis; *GPAM* (*P* = 0.035), *MDH1* (*P* < 0.001), fatty acid transport; *FABP1* (*P* = 0.008), *ACLY* (*P* < 0.001), and fatty acid oxidation; *ACADL* (*P* = 0.015), *CPT-2* (*P* = 0.019) were observed for broilers. Layers and cross comparisons indicated no real differential expression in fatty acid metabolism between the groups, with the exception of down regulation of lipogenic gene *SCD1* (*P* = 0.003) and fatty acid oxidation *CPT-2* (*P* < 0.001) and *ACAA1* (*P* < 0.001) genes. Layers in comparison to the cross also had upregulated expression of the transcription factor *PPARA* (*P* = 0.047), a difference not seen elsewhere.

**Table 5:** Pearson correlation coefficient (*r*) of target gene against individual bodyweight (BW), and mean expression levels (CPM) of genes between broilers (*n* = 6), cross (*n* = 6) and layers (*n* = 6)

Gene name	*r* (Gene vs. BW)^1^	Broiler (*n* = 6)	Cross (*n* = 6)	Layer (*n* = 6)	Regulation^2^
*ACACA*	0.695^**^	3135.4 ± 118.6^a^	2918.5 ± 101.4^a^	2367.5 ± 135.4^b^	↑
*ACADL*	0.734^**^	751.3 ± 27.8^a^	648.1 ± 15.3^b^	620.9 ± 23.4^b^	↑
*ACLY*	0.855^**^	3605.7 ± 201.6^a^	2386.9 ± 117.4^b^	1956.9 ± 163.3^b^	↑
*ACSL1*	0.336	693.5 ± 61.9	553.0 ± 21.6	612.8 ± 27.1	
*APOA1*	0.639^**^	1519.0 ± 107.2^a^	1348.5 ± 42.2^ab^	1194.1 ± 57.3^b^	↑
*APOC3*	0.736^**^	1859.5 ± 131.2 ^a^	1472.7 ± 63.2^b^	1307.6 ± 77.4^b^	↑
*CD36*	0.593^**^	517.8 ± 24.6^b^	580.4 ± 15.1 ^ab^	596.5 ± 15.8^a^	↓
*CPT1A*	0.044	244.5 ± 37.1	247.6 ± 15.5	233.5 ± 10.0	
*CPT2*	0.853^**^	224.7 ± 8.4 ^a^	195.4 ± 6.7^b^	151.8 ± 4.3^c^	↑
*FABP1*	0.722^**^	998.8 ± 96.5^a^	687.5 ± 30.3^b^	606.7 ± 38.4^b^	↑
*FADS6*	0.547^*^	109.6 ± 8.3	130.4 ± 11.7	145.1 ± 9.1	↓
*FASN*	0.769^**^	10,794 ± 755.5^b^	8475.9 ± 480.1^a^	6486.9 ± 559.1^a^	↑
*LPL*	0.600^**^	48.2 ± 22.3^b^	90.1 ± 9.1 ^ab^	117.9 ± 9.6^a^	↓
*MDH1*	0.902^**^	667.0 ± 28.0^a^	462.6 ± 18.8^b^	386.7 ± 12.3^b^	↑
*ME1*	0.601^**^	1045.0 ± 127.5^a^	963.7 ± 75.0^ab^	600.6 ± 92.9^b^	↑
*PPARA*	0.376	447.1 ± 17.3 ^ab^	434.6 ± 17.1^b^	496.8 ± 15.3^a^	
*RXRA*	0.012	65.9 ± 2.9	63.4 ± 2.8	64.6 ± 3.0	
*SCD*	0.817^**^	2785.2 ± 130.0^a^	2322.6 ± 81.9^a^	1413.6 ± 233.1^b^	↑
*TLR2A*	0.041	21.1 ± 1.3	20.9 ± 1.9	20.4 ± 1.7	
*TLR4*	0.360	10.2 ± 0.9	9.3 ± 0.4	12.6 ± 1.2	
*XBP1*	0.620^**^	225.6 ± 9.1^b^	231.4 ± 9.9^b^	281.8 ± 12.4^a^	↓
*ERN1*	0.578^*^	28.9 ± 2.5	23.9 ± 1.2	23.2 ± 1.1	↑

^1^Pearson’s correlation coefficient of target gene against individual bodyweight of all three groups of birds (BW); ^*^Sig at *P* < 0.05, ^**^Sig at *P* < 0.01

^2^Relative direction of regulation: ↑ Broiler upregulated (broiler > cross > layer); ↓Broiler downregulated (broiler < cross < layer)

^a-c^Means (± SEM) within the same row for each parameter with different superscripts are significantly different (*P* < 0.05). Means values are counts per million (CPM) transcripts, to correct for varying sequence depth between individual samples

Endoplasmic reticulum (ER) stress-related gene *ERN1* was not differentially expressed between any of the three groups (*P* = 0.67). *XBP1* was found to be significantly upregulated in layers in comparison to both broilers (*P =* 0.002) and crossed birds (*P* = 0.007). Toll-like receptors *TLR2* and *TLR4* were not found to be differentially expressed between any of the three groups (*P* = 0.951).

Pearson’s two-tailed correlations with individual bird bodyweights (Table 5), revealed 15 of the 22 genes were highly correlated with bodyweight at *P* < 0.01, 2 genes correlated at *P* < 0.05 and 6 of the genes non-significant with bodyweight. The highest correlation was between malate dehydrogenase (*MDH1*) and bodyweight (*r* = 0.902; *P* < 0.001).

## Discussion

The aim of this experiment was to elucidate how genetic selection has influenced carcass composition, fatty acid metabolism and select innate immune parameters. The objective was to further develop the understanding of factors which may be underpinning performance variation in modern broilers. Our previous experimental work did not provide sufficient phenotypic variation in feed conversion ratio within flock, thus it was decided to investigate birds with grossly different growth potentials; namely, broilers, layers and a layer × broiler F1 cross. Although samples were taken at multiple time points, d 14 was selected as the primary sampling date due to the rapid growth acceleration seen in broilers from 2 to 3 wk of age. By sampling at this time point it was hoped to capture physiological changes at the beginning of the growth acceleration to further understand broiler growth rates.

As expected the growth rates of the broiler progeny well exceeded those of the layer strain progeny. By d 14 the broilers were four times the weight of the layer strain males and twice the weight of the F1 cross. The total lipid carcass percentage of the broilers was higher than both the layers and the cross, which weren’t significantly different from each other, despite the cross being twice the weight of the layers. Interestingly multiple studies have shown that the dietary fatty acid composition is reflected in the fatty acid composition of the tissues and serum of broilers [33, 34]. Despite being raised in the same environmental conditions and fed the same diet, the fatty acid composition of the carcasses and blood spots differed between the three groups in this study, suggesting difference existed in fatty acid metabolism. The broilers had increased overall MUFA percentages, which would correlate with the significant upregulation of *SCD1* which encodes the rate-limiting enzyme converting SFAs into MUFAs [35]. Comparisons of the total SFA, MUFA and PUFAs revealed layers had higher n-6 and n-3 levels, indicating two possibilities, layer strains either have a higher physiological requirement for long chain PUFAs, or, layers are more efficient at converting available dietary linoleic and alpha-linolenic fatty acids to their long chain derivatives. The gene encoding the enzyme *FADS6*, which is rate limiting in the elongation of PUFAs, was found to be upregulated in the layers in comparison to the broilers which may support this concept.

Whilst it may be anticipated that the increased fat deposition is due to either increased lipogenesis and/or a decrease in fatty acid *β*-oxidation, we saw a net overall increase in both lipogenesis and fatty acid *β*-oxidation genes in the broilers compared to layers or the F1 cross. Although this could be controlled by transcription factors regulating FA metabolism, such as the nuclear receptor *PPARA*, we found no evidence to support this. The higher metabolic activity may therefore be reflective of the weight of the liver at d 14 which was relatively larger than that of the layers expressed as a percentage of bodyweight. An early increase in liver mass has also been observed in multiple studies, including comparisons of modern broilers and heritage lines [2]. In the current study the layer and crossed birds had reached their peak relative liver mass by d 7, however the broilers had higher relative weights at d 7 and reached their relative maximum weights at d 14 post hatch. By d 28 there were no differences in relative liver mass between the broilers, layers and their F1 cross. Schmidt et al., [2] propose this early increase in liver mass could correspond to increased liver capacity required in early post hatch, and that a possible effect of selection may have shifted earlier maturation of the liver in modern broiler lines. The relative heart weights followed a similar pattern to the liver in that they were at their maximums in the first 2 wks post hatch. From d 14 onwards the broiler relative heart weights had significantly reduced when compared to the cross and layers. These findings are not surprising as the reduction in cardiac relative size and capacity has been well documented in broilers due to genetic selection for increased growth [2, 18, 36].

Additional to differential fatty acid metabolism, it was hypothesised that innate immune parameters may also be interacting with fatty acid metabolism ultimately influencing performance variation. Modern broilers are now considered obese relative to layer strains, so obesity-related pathologies such as inflammation and cellular stress may be anticipated to be increased in broilers. To test this hypothesis immune organ weights (spleen and bursa), heterophil: lymphocyte ratios, as well as Toll-like Receptors (*TLR2a, TLR4*), fatty acid translocase (*CD36*) and endoplasmic reticulum stress indicator genes (*ERN1, XBP1*) were included in the current study.

The relative weight of both the spleen and bursa continued to increase in the cross and layer birds from d 0 until d 28 post hatch. The broilers reached maximum relative spleen and bursa weights at d 14 and then decreased from there on in. There has been conflicting interpretation as to whether relative increased immune organ size equates to a better immune defence system. Once such study found that the size of the spleen for example was correlated with changes in body condition, and that size was elevated in individual birds in prime body condition [37]. It could be argued that all of our birds were in good body condition, as there was no disease, parasite infection or mortality. Body condition as measure of fatness v leanness however, as used by Møller et al., [37], would assume the layers and the F1 cross were in better relative condition than the broilers, and potentially reflective of the smaller immune organs. Additionally broilers have repeatedly been shown to be less responsive to immune challenges experimentally, and this has been attributed to a negative consequence of genetic selection [24]. Although relative decreased lymphoid organ weights (% of bodyweight) were observed in the broilers compared to the cross and layers, there was no evidence to suggest that the broilers were compromised immunologically due to increases in fat deposition in an unchallenged experimental setting. Heterophil: lymphocyte ratios were not significantly different between any of the birds although there was a high level of variation between the individuals. The cross did appear to have a lower ratio, however this is more likely attributed to a lower number of samples and the high variation in individual birds than a significant trend.

Whilst short-term stress is of minimal consequence to broilers, long-term stress results in increased serum corticosterone, increased heterophil: lymphocyte ratios and altered protein, carbohydrate and lipid metabolism, and increased deposition of abdominal fat [38]. This poses a question, could a broiler be chronically stressed at a cellular level, particularly with the reduction of organ weights relative to overall bodyweight as growth increases? To investigate whether there was any evidence of organelle stress occurring, two key ER stress indicators which initiate the unfolded protein response (UPR) were assessed, inositol-requiring kinase 1 (*ERN1*) and x-box binding protein (*XBP1*). Saturated fatty acids have been shown to trigger the UPR response in hepatocytes and the UPR has been linked to lipid synthesis and breakdown [39]. Despite the broiler, layer and F1 cross birds having differing SFA levels, no differences were found in the expression levels of *ERN1*, *XBP1* however was found to be upregulated in the layers in comparison to both the broiler and cross. Given that *ERN1* levels are showing no indication of ER stress, the differential expression of *XBP1* may align with the suggestion that *XBP1* functions as a mediator of hepatic lipogenesis, distinct from its function in ER stress and the UPR [40]. It is thought to regulate the transcription of genes involved with fatty acid synthesis, including *SCD1* and *ACACA*, with deletion of *XBP1* resulting in decreased triglyceride, cholesterol and free fatty acids [40]. It is difficult to conclude whether *XBP1* is exhibiting a regulatory effect on lipogenesis in the layers however the aforementioned genes are not seen to be increased in the layers compared to the broilers or the cross.

Additional to organelle stress, Toll-Like receptors, including *TLR2* and *TLR4* have received attention for their roles in the development of obesity and insulin resistance, although the mechanisms by which they contribute still remain unclear. Mice lacking *TLR2* and *TLR4* genes do show however that TLRs are involved in the development of obesity [41]. In macrophage cell cultures, saturated fatty acids, such as stearic acid and palmitic acid, have been shown to activate *TLR2* and *TLR4* signalling pathways, which consequently activates down steam pro-inflammatory pathways, Conversely, PUFAs, particularly n-3 s, have been shown to inhibit TLR2/4 expression, activation and downstream signalling [42]. In our current study we found no differential expression of *TLR2a* in the avian liver in any of the three types of birds. Additionally we found no evidence in the expression levels of *TLR4* to suggest that the differing fatty acid profiles of the birds was having an effect or interaction with the expression of *TLR4* at d 14 post hatch. This was also the case for *CD36*, with the exception of a down regulation in the broilers in comparison to the layers. Given the biological diversity for the role of *CD36*, this likely does not translate into down regulated facilitation of fatty acid transport given the overall upregulation of fatty acid metabolism seen in the broilers.

## Conclusion

The results indicate a total upregulation of fatty acid metabolism in broiler chickens when compared to an F1 cross and commercial layer strain. This increase is most likely as a result of genetic selection for growth, with the overall increase resulting in increased FA synthesis as well as *β*-oxidation in the liver. There was no evidence to suggest that at d 14 post hatch that the broilers are in a state of cellular hepatic stress or demonstrating changes in innate immunity parameters such as *TLR2* and *TLR4* expression. This is despite the broilers growing at four times the rate of the layers and with significant increases in fat %. Day 14 post hatch was selected to capture the physiological changes as the broiler growth acceleration begins. It is possible that the d 14 sample time point was too early in relation to fatty acid metabolism and innate immunity/cellular stress interactions to capture changes that may ultimately be driving performance. Analysis at additional time points in the grow out phase could better revel indicators of chronic stress as the organ weights continue to decrease by relative weight, contributing to metabolic stress and altering metabolism.
